# DP-b99 Modulates Matrix Metalloproteinase Activity and Neuronal Plasticity

**DOI:** 10.1371/journal.pone.0099789

**Published:** 2014-06-11

**Authors:** Marine Yeghiazaryan, Izabela Rutkowska-Wlodarczyk, Anna Konopka, Grzegorz M. Wilczyński, Armenuhi Melikyan, Eduard Korkotian, Leszek Kaczmarek, Izabela Figiel

**Affiliations:** 1 Department of Neurophysiology, The Nencki Institute of Experimental Biology, Warsaw, Poland; 2 Department of Molecular and Cellular Neurobiology, The Nencki Institute of Experimental Biology, Warsaw, Poland; 3 D-Pharm Ltd, Kiryat Weizmann Science Park, Rehovot, Israel; 4 Department of Neurobiology, Weizmann Institute, Rehovot, Israel; National Center for Scientific Research Demokritos, Greece

## Abstract

DP-b99 is a membrane-activated chelator of zinc and calcium ions, recently proposed as a therapeutic agent. Matrix metalloproteinases (MMPs) are zinc-dependent extracellularly operating proteases that might contribute to synaptic plasticity, learning and memory under physiological conditions. In excessive amounts these enzymes contribute to a number of neuronal pathologies ranging from the stroke to neurodegeneration and epileptogenesis. In the present study, we report that DP-b99 delays onset and severity of PTZ-induced seizures in mice, as well as displays neuroprotective effect on kainate excitotoxicity in hippocampal organotypic slices and furthermore blocks morphological reorganization of the dendritic spines evoked by a major neuronal MMP, MMP-9. Taken together, our findings suggest that DP-b99 may inhibit neuronal plasticity driven by MMPs, in particular MMP-9, and thus may be considered as a therapeutic agent under conditions of aberrant plasticity, such as those subserving epileptogenesis.

## Introduction

DP-b99[1,2-Bis(2-aminophenoxy)ethane-N,N,N’N’-tetraaceticacid, N,N’-di(octyloxyethyl ester), N,N’-disodium salt, CAS Number: 222315-66-4] is a lipophilic, cell permeable diester, derivative of BAPTA that is able to selectively chelate transition metals such as zinc, copper, and iron within membranes [1]. DP-b99 was initially developed by D-Pharm Ltd. as a neuroprotectant for acute ischemic stroke [2] and preclinical studies with radiolabeled DP-b99 in rats indicate that DP-b99 penetrates the rodent brain. This promising compound was undergoing evaluation in phase III clinical trials [3], but recent studies revealed lack of neuroprotective capacity for DP-b99 under those conditions [4]. However, despite the phase III MACSI trial failure, the drug may yet be efficacious in other indications.

DP-b99 was previously shown to prevent zinc-induced neuronal cell death [5]. Zinc is considered as a key mediator and modulator of the neuronal death associated with transient global ischemia and sustained seizures [6], [7]. It was demonstrated that DP-b99 effectively attenuates microglial activation and diminishes the activity of zinc-dependent matrix metalloproteinase 9 (MMP-9) and tumor necrosis factor-α (TNF-α) - converting enzyme *in vitro* and *in vivo* [1], [8], [9]. Thus, it seems to be important to evaluate efficacy of DP-b99 in blocking MMP-mediated processes in the brain.

Matrix metalloproteinases (MMPs) are a family of secreted and transmembrane endopeptidases that exert their function through cleavage of proteins of the extracellular matrix. All MMPs are active at neutral pH, require calcium for activity and contain a zinc atom within the catalytic domain [10]. MMPs are involved in numerous physiological phenomena, such as development, cell migration, learning and memory [11], [12]. They are also implicated in various pathophysiological processes and subsequent regenerative attempts, including cancer, epilepsy, stroke, inflammation, neurodegeneration and wound healing. Notably, one type of MMP, MMP-9, has recently been recognized as contributing to learning and memory, as well as synaptic plasticity, at the physiological and morphological levels [13], [14], [15], [16], [17].The association of MMP-9 with neuroplasticity has also been supported by studies involving MMP-9 knockout mice and transgenic rats overexpressing the enzyme. For instance, MMP-9 knockout mice showed decreased sensitivity to chemical kindling [18], [19], whereas transgenic rats overexpressing MMP-9 developed enhanced seizure progression [18].

Considering all of the above, we therefore postulate that the pharmacological inhibition of MMPs might be beneficial by suppressing seizure progression in response to chemical kindling. Kindling (repeated chemical stimulation of the brain, which inhibits GABAergic transmission in CNS) is widely accepted as a functional model of neuronal network activity, in which the altered neuronal response develops in the absence of gross morphological damage [20].

## Materials and Methods

### Animals

A total of 14 wild-type male C57Bl/6 mice (3 months old at the start of the experiment), 25–30 g, were used in the present study. All animals were housed in the same facility with a 12∶12 h light-dark cycle in the animal house of the Nencki Institute of Experimental Biology. The experimental protocols were approved by the Local Ethical Committee on Animal Experiments of the Nencki Institute (Permit Number: 174/2011) and all efforts were made to minimize animal suffering and to decrease the number of animal used.

### PTZ Kindling

Animals received a dose of 35 mg/kg PTZ (Sigma-Aldrich) intraperitoneally (i.p.), once every 48 hours till mice were kindled and sacrificed. After each PTZ injection, animals were placed into chambers and behavioral seizure activity was recorded within 30 min after administration. Seizure activity was classified according to the following scale (adapted from [21]: 0, no convulsive behavior; 1, facial movements; 2, rhythmic head movements, head nodding; 3, unilateral forelimb clonus; 4, bilateral forelimb clonus and rearing; 5, falling and clonic convulsions; 6, death. Mice were considered kindled when seizures at score 4 and 5 occurred after each PTZ injection for three consecutive days. Animals were sacrificed 5 min after the seizure onset and brains were used for further analysis.

### Effect of DP-b99 on PTZ Kindling Induced Seizures

Fourteen animals were divided into a control (vehicle treated) and DP-b99 group, each containing 7 mice. The mice of both groups were treated every second day with PTZ i.p. injection (35 mg/kg). Mice of the DP-b99 group were administered 0.3 mg/kg/day of DP-b99 (D-Pharm Ltd, Rehovot, Israel) i.p. once every 24 hours, 3 hours before PTZ injection, while animals of the PTZ group at the same time received an injection of vehicle (appropriate volume of PBS, pH 7.4). DP-b99 was dissolved in PBS, pH 7.4. Solutions were freshly prepared every day.

### Immunohistochemistry and Quantification

Kindled mice (n = 4 per group) were sacrificed by decapitation immediately after isoflurane anesthesia. Brains were rapidly removed, divided into 2 equal hemispheres. One hemisphere was used for hippocampus isolation, which was rapidly frozen in dry ice for later Western blot analysis. The second hemisphere was fixed in 4% PFA for 2 days before embedding in paraffin. Then, 10-µm -thick sections were cut coronally on a microtome and collected on glass slides. Tissue sections were deparaffinized in xylen, rehydrated in graded ethanol series and moved to antigen retrieval for 20 min in a citrate buffer. The washed sections were blocked with 5% normal donkey serum in 0.1% PBS-Triton X-100 (PBST) for 60 min at room temperature to reduce non-specific staining. Then, sections were labeled overnight at 4°C with zinc transporter-3 antibody (ZnT-3; 1∶500, Synaptic Systems) in NDST. This antibody is specific for ZnT-3 and has been shown to selectively stain mossy fibers and its terminals [22]. Before incubation with secondary antibody, sections were washed three times for10 min each in PBS containing 0.1% Triton X-100 at room temperature. Secondary antibody conjugated with a range of Alexa fluorophore dyes (Molecular Probes) diluted in NDST was applied for 2 h at room temperature. Sections were washed out with PBS containing 0.1% Triton X-100 and DPX was used as mounting reagent.

Mossy fiber projections were visualized by ZnT-3 immunofluorescence labeling and quantified using ImageJ software. At least four brain sections from each mouse were analyzed. To assess mossy fiber sprouting, the length of infrapyramidal mossy fiber bundle (IP) was normalized to that of the suprapyramidal (SP), as was previously described [23]. Briefly, to determine IP length a line extending from the dentate hilus to where the mossy fibers travel below the pyramidal cell layer was drawn as a guide and measured. The length of SP was measured from the same point to the apex of the curvature of the CA 3 pyramidal cell layer. From these data the ratio IP : SP was calculated.

### Western Blot

To assess the β-dystroglycan (β-DG) cleavage [24], the hippocampi, obtained from control and DP-b99 groups (n = 4 per group), were homogenized in ice-cold buffer (50 mM Tris-HCl, pH 7.5; 150 mM NaCl; 5 mM CaCl_2_ and 10 mg/ml protease inhibitor mixture, Sigma). The protein concentration was spectrophotometrically determined in the fractions using BCA protein assay reagent (Thermo Scientific). Twenty micrograms of total protein samples were mixed with reducing Laemmli sample loading buffer, denatured and separated on a 10% SDS-polyacrylamide gel at 100 V for 2 h. The resolved proteins were then transferred to PDVF membranes at 350 mA for 2 h and then soaked in 2.5% non-fat milk in Tris-buffered saline containing 0.1% Tween 20 (TBST) for 1 h at room temperature. After blocking, the membranes were incubated overnight at 4°C with anti-β-dystroglycan antibody (Novocastra, 1∶500) diluted in 2.5% non-fat milk in TBST. The blots were washed 3 times (15 min per wash) with 5–10 ml of TBST followed by 2 h incubation with peroxidase-conjugated secondary antibody (Amersham), diluted 1∶10000 in TBST containing 2.5% non-fat milk. After washing of secondary antibody, the bands were detected using a chemiluminescence detection system, ECL plus (Amersham). Then the blots were stripped and re-probed with anti-β-actin antibody (Sigma, 1∶12000). Relative signal densities representing amounts of β-DG were measured densitometrically, and normalized against β-actin.

### Enzymatic Assay Using DQ-gelatin

To test for the DP-b99 inhibitory effect against gelatinase activity we employed EnzChek gelatinase/collagenase assay kit (Molecular Probes), where dye quenched (DQ) gelatine is used as a substrate for MMP-9. DQ gelatin is heavily labeled with FITC molecules and the fluorescence is quenched. After being cleaved, the fluorescence is enhanced and can be quantified by a fluorescence microplate reader (excitation 495/emission 515 nm). The assay used Molecular Devices Spectra Max M5e. The procedure was followed according to manufacturer’s instructions with some modification (the reaction buffer used in experiments contained 5 µM Ca^2+^ instead of 5 mM). DQ gelatine was incubated with DP-b99 (0.12 µM or 20 µM) and human recombinant auto-activating MMP-9 (400 ng/ml) at 37°C. Fluorescence was measured every minute for up to 60 min. The general MMP inhibitor GM6001 (25 µM) and specific Inhibitor I (Calbiochem) of MMP-9 and MMP-13 (5 µM) were used as positive controls. Each experiment was repeated three times. Data were normalized and for each time point background fluorescence was corrected by subtracting the values of non-enzymatic control.

### Organotypic Hippocampal Cultures and Kainate Treatment

Cultures of hippocampal slices were prepared according to the method of Jourquin et al. [25], with slight modifications. Briefly, 7 day-old Wistar rat pups were sacrificed in accordance with institutional guidelines and as approved by the Animal Care and Use Committee. The brains were removed and placed in cold (4°C) Gey’s balanced salt solution (Sigma) supplemented with glucose (6 mg/ml). The hemispheres were separated and individual hippocampi were isolated and immediately cut into 350 µm thick slices using a McIlwain tissue chopper. Slices were transferred to Millicell inserts (Millipore, CM) with three-four slices per insert and placed in six-well tissue culture plates. Cultures were grown in 1 ml of maintenance medium (50% minimum essential medium, 25% Hank’s balanced salt solution, 25% horse serum, 25 mM glucose, 2 mM glutamine) at 37°C in a 5% CO_2_/95% O_2_ air humidified incubator with complete medium changes twice per week. Viability in the slices was determined using the fluorescent marker, propidium iodide (PI), which stains only dying cells from damaged cell membranes. PI (5 µg/ml) was added to the medium 1 day before experiment. After 14 days *in vitro* (DIV) the cultures were treated with 5 µM kainic acid (KA, Sigma) for 24 h. DP-b99 was dissolved in absolute ethanol/5% bovine serum albumin and added to slice cultures at 1/10 medium volume (100 µl/well) 1 h before KA. We used two different final concentrations of DP-b99∶20 µM and 0.12 µM. The higher dose has been reported to be effective at protecting neurons against a toxic Zn^2+^-rise [5]. The cultures were then fixed and processed for morphological analysis as previously described [26].

### Dissociated Hippocampal Cultures

Hippocampal cultures from P0 Wistar rats were prepared as previously reported [27]. Cells were plated at a density of 100,000 cells per 18 mm diameter coverslip coated with 1 mg/ml poly-L-lysine (Sigma) and 2.5 µg/ml laminin (Roche). At 10 DIV cells were transfected using Effectene (Qiagen) according to the manufacturer’s protocol with plasmid carrying eGFP under the β-actin promoter. After 20 DIV cultures were incubated for 1 h with 400 ng/ml of auto-activating MMP-9. Some of the cultures were pre-treated for 1 h with DP-b99 (0.12 µM or 20 µM) or GM6001 (25 µM). Cells were fixed in 4% paraformaldehyde (PFA) in PBS, permeabilized with 0.1% Triton X-100 in PBS and incubated for 1 h with Alexa Fluor 568-coupled phalloidin (1∶50; Molecular Probes). After rinsing with PBS, coverslips were mounted on glass slides with Vectashield (Vector Laboratories). Confocal microscopy was performed using a Leica TCS SP5 confocal microscope with PL Apo 40×/1.25 NA oil immersion objective using a 561 nm line of diode pumped solid state laser at 10% transmission at a pixel count of 1024×1024. A series of z-stacks were acquired for a cell with step 0.4 µm. The images were analyzed semi-automatically using the custom written software (SpineMagick software, patent pending EP 11461530.5), as previously described [28]. Briefly, to determine the spine length, we measured the curvilinear length along the spine virtual skeleton, which was obtained by fitting the curve (the forth degree polynomial). F-actin staining was expressed quantitatively as background corrected ratio of fluorescence intensity of Alexa Fluor 568 coupled to phalloidin, calculated within spines vs. dendritic shaft.

### Statistical Analysis

Data are presented as mean ± standard error (SE) of the mean. Student’s t-test, and repeated measures ANOVA were used to compare the statistical significance of differences between control and treated groups. Values of p<0.05 were deemed significant.

## Results

### DP-b99 Inhibits Development of PTZ-induced Kindled Seizures

**Figure 1:**
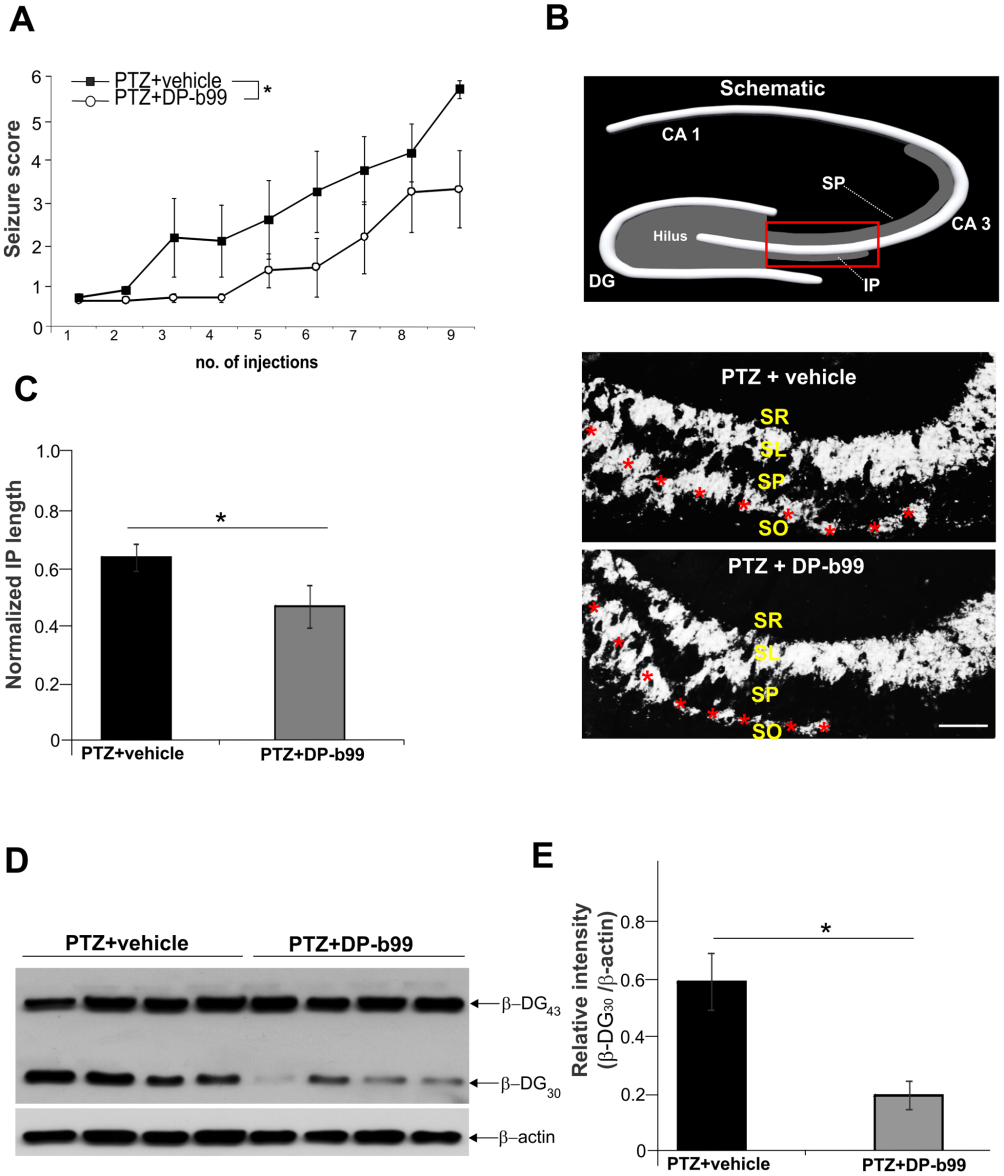
Effect of DP-b99 on PTZ kindling induced seizures. **A**- Seizure scores of PTZ-kindled vehicle- and DP-b99 treated mice during the course of the experiment (mean ± SE). Note the delay of seizures in DP-b99 treated group (n = 7 per group, repeated measures ANOVA:F (1, 10) = 5.6382, p<0.05). **B** – Schematic diagram of a hippocampal section showing suprapyramidal (SP) and infrapyramidal (IP) bundles of mossy fiber pathway arising from hilus of dentate gyrus (DG) to area of CA 3 pyramidal cells. Representative images of coronal sections immunostained for ZnT-3 (white) in CA3 hippocampal region of vehicle- and DP-b99 treated kindled animals. Vehicle treated animals demonstrated much longer IP length, indicating a robust mossy fibers sprouting (red asterisks). SR, stratum radiatum; SL, stratum lucidum; SP, stratum pyramidale; SO, stratum orient. Scale bar = 100 µm. **C**– Quantitative analysis of Zn-T3 immunofluorescence in vehicle- and DP-b99 treated kindled animals. ZN-T3 expression was evaluated in IP mossy fibers using the ratio of IP length to the length of SP (n = 4 animals for each group, mean ± SE; * - indicates p<0.05). **D, E -** Western blot analysis and quantification of β-dystroglycan cleavage product in the hippocampus of kindled animals (n = 4 per group, mean ± SE; * - indicates p<0.05).

We investigated the effect of DP-b99 on epileptogenesis by employing the mouse PTZ kindling model. The animals were injected with 35 mg/kg of PTZ, every second day, to evoke seizure development and, in addition, were pretreated with either DP-b99 or vehicle. We found that daily administration of DP-b99, 3 h prior to PTZ injection attenuated the sensitivity of mice to PTZ and significantly delayed the development of PTZ-induced kindled seizures (repeated-measures ANOVA: F (1, 10) = 5.6382, p<0.05; Fig. 1 A).

Sprouting of mossy fibers might underlie PTZ kindling (see e.g., Wilczynski et al. [18]). To determine whether the delay of PTZ-induced seizures in the DP-b99 treated mice group might correlate with diminished sprouting, we performed ZnT-3 immunostaining of the brain sections obtained from vehicle and DP-b99 treated animals. The mossy fiber axons of dentate gyrus granule cells form two bundles (SP and IP), which extend from the dentate hilus to area of CA 3 pyramidal cells (see schematic diagram, Fig. 1 B). We found that vehicle treated animals have longer IP length, indicative of robust mossy fiber sprouting and resulting in the formation of new synapses on the basal dendrites of the stratum oriens of the CA 3 pyramidal cells (Fig. 1 B). The evaluation of Zn-T3 expression in IP mossy fibers of PTZ kindled animals, revealed that consecutive daily administration of DP-b99 results in significantly less mossy fiber sprouting in the CA 3 area than in vehicle treated mice(p<0.05; Fig. 1 C).

Previously, MMP-9 has been specifically implicated in PTZ kindling [18], [19] and Michaluk et al. [24] have shown that β-dystroglycan (β-DG) is selectively cleaved by MMP-9 in response to enhanced neuronal activity, providing very sensitive measure for the enzyme (see also Knapska et al. [29]). Therefore, to determine the effect of DP-b99 treatment on MMP-9 activity, proteolysis of β-DG was evaluated by western blotting. In all eight kindled mice (n = 4, per group) the cleavage product of β-DG (30 kDa) was detected. However, in animals subjected to the kindling and in addition treated with DP-b99, markedly diminished cleavage was observed (Fig. 1 D). Densitometric quantification revealed that DP-b99 treatment significantly reduced the presence of the β-DG cleaved form in kindled mice in comparison with PTZ-kindled animals that received vehicle (Fig. 1 E).

### DP-b99 Inhibits Gelatinolytic Activity

**Figure 2:**
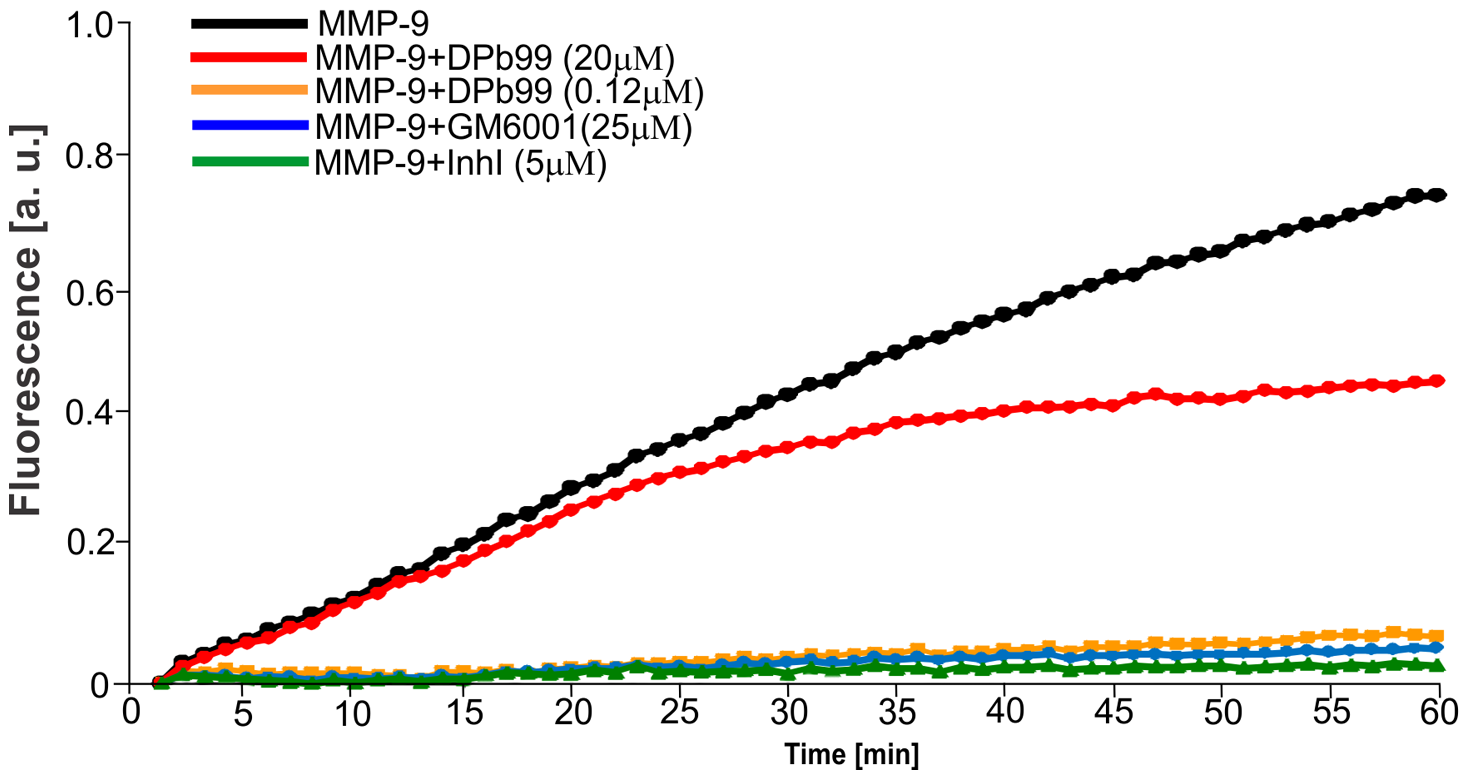
Enzymatic assay using DQ-gelatine. 400/ml of purified MMP-9 was incubated with DQ-gelatine and with DP-b99 of different concentrations (0.12 µM and 20 µM) at 37°C. Fluorescence was measured every minute. General MMP inhibitor GM6001 (25 µM) and specific Inhibitor I of MMP-9 and MMP-13 (5 µM) were used as a positive controls.

Results of the previous experiment suggested that MMP-9 can be targeted by DP-b99. Therefore, we examined whether DP-b99 inhibits MMP-9 activity *in vitro*. We used a fluorometric gelatinolytic assay, in which recombinant MMP-9 as well as its known inhibitors (GM6001 and Inhibitor I) were employed (see also Michaluk et al. [27]). Fig. 2 shows that DP-b99 at 20 µM, and even more robustly at 0.12 µM, exerts inhibitory activity against MMP-9.

### DP-b99 Diminishes the Neurodegenerative Effect of Kainate

**Figure 3:**
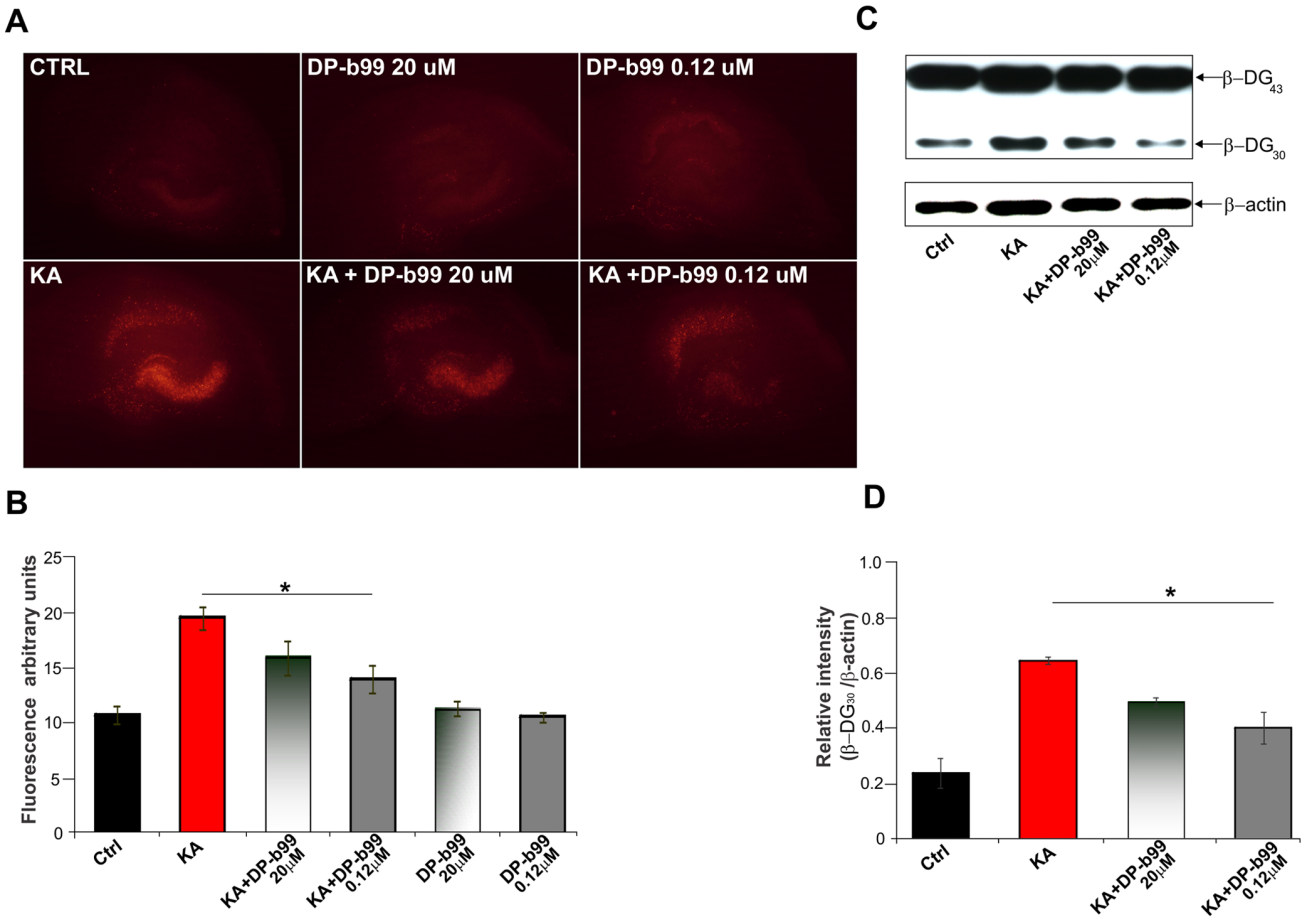
DP-b99 diminishes neurodegenerative effect of kainate in hippocampal slice cultures. **A** – Representative fluorescence photomicrographs of organotypic hippocampal slices (16 DIV) showing uptake of PI 24 h after various treatments: CTRL (untreated cultures); DP-b99 20 µM and 0.12 µM; KA (5 µM); KA+DP-b99 20 µM or 0.12 µM. **B -** Quantification done by ImageJ software and represented in arbitrary units of fluorescence shows a significant decrease in neuronal death in all groups treated with DP-b99 (n = 3, mean ± SE; * - p<0.05). **C -** Western blot analysis of β-dystroglycan cleavage. Organotypic hippocampal cultures were treated with 50 µM KA for 10 min. DP-b99 (0.12 µM or 20 µM) was added to the culture media 1 h before KA administration. **D –** Quantification of β-dystroglycan 30 kDa product of MMP-9 cleavage intensity from three independent experiments (n = 3, mean ± SE; * - p<0.05).

Previously, it was shown that MMP-9 plays a crucial role in neuronal cell loss evoked by kainate (KA) in the hippocampal organotypic slice cultures [25], [26].Therefore, we decided to study the DP-b99 effects on this phenomenon. The hippocampal organotypic cultures were stained with PI to show disrupted cellular integrity (see also Mioduszewska et al. [30]). Indeed, the staining revealed enhanced neuronal damage in the slices treated with KA in comparison to control, DP-b99 alone or vehicle treated cultures, whereas pretreatment with DP-b99 led to less pronounced excitotoxicity (Fig. 3 A, B). To see whether MMP-9 is also inhibited under these culture conditions, we have examined the effect of DP-b99 on KA evoked cleavage of β-DG. As shown in Fig. 3 C, the immunoblot analysis of the cultures stimulated with 50 µM KA for 10 min revealed a significant increase in the level of the cleaved, 30 kDa form of β-DG, whereas 1 h pretreatment of the cultures with DP-b99 effectively prevented β-DG proteolysis (Fig. 3 D).

### DP-b99 Suppresses MMP-9 Influence on Dendritic Spine Morphology

**Figure 4:**
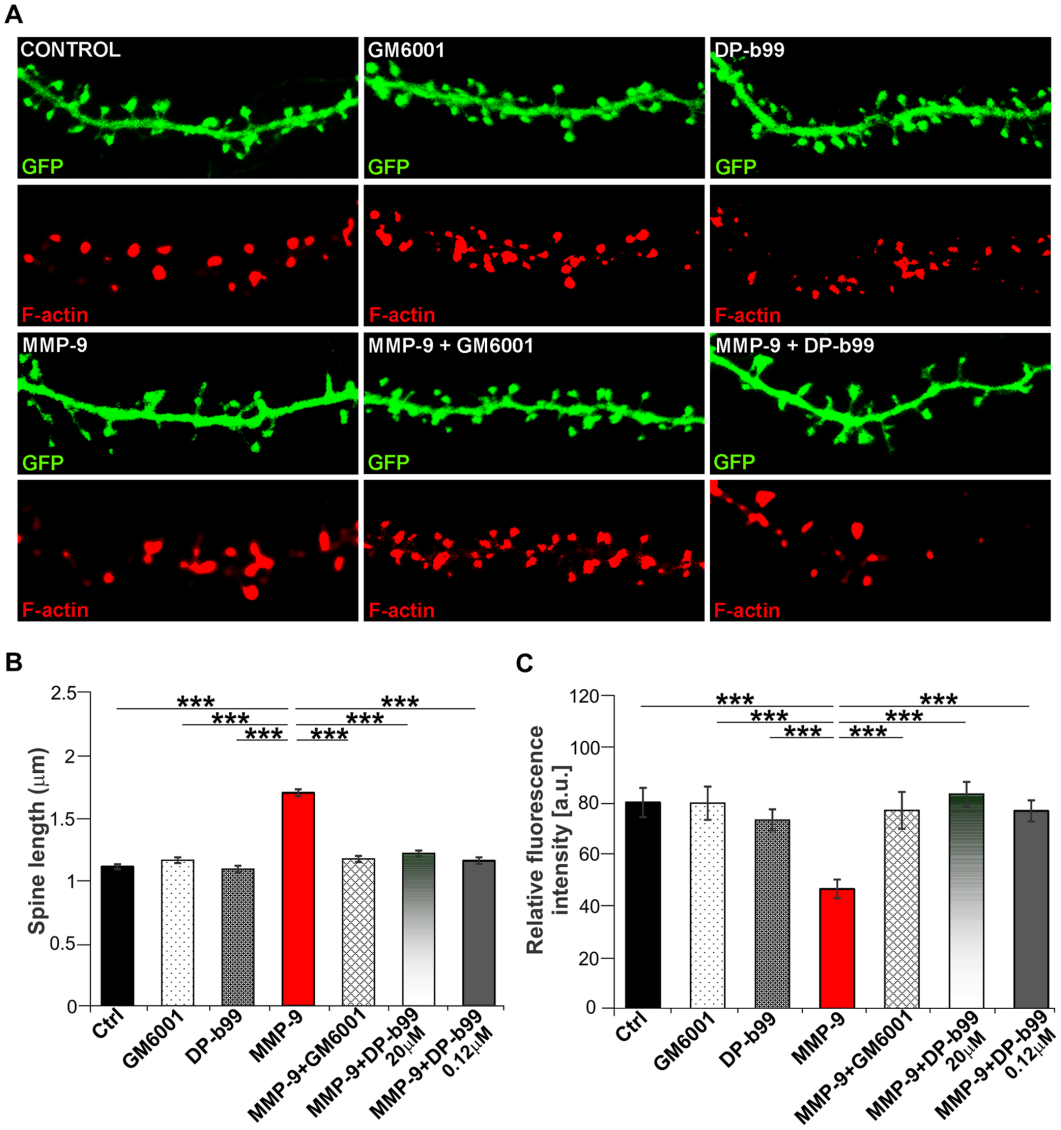
DP-b99 prevents MMP-9 effect on dendritic spine morphology and F-actin arrangement. **A** - Representative dendrites of GFP-transfected hippocampal neurons showing morphological changes in spines after indicated stimulations. Lower panels show the same dendrites following staining with Alexa Fluor 568 coupled to phalloidin (F-actin). Note that 1 h incubation of the cultures with MMP-9 (400 ng/ml) promotes dendritic spine elongation, whereas 1 h pretreatment with DP-b99 (0.12 µM) prevents the formation of these filopodia-like structures. Neither GM6001 nor DP-b99 on their own evoke spine elongation. **B -** Relative changes in spine length following 1 h stimulation with MMP-9 with or without DP-b99 or GM6001 pretreatment (n = 3, mean ± SE; *** - p<0.001). **C -** Fluorescence intensity of Alexa Fluor 568 coupled to phalloidin, calculated within spines vs. dendritic shaft (n = 3, mean ± SE; *** - p<0.001).

Previous studies have shown that MMP-9 is involved in synaptic plasticity through influence on dendritic spine morphology, especially by provoking spine elongation [27]. Hence, we tested whether DP-b99 might reverse this effect in dissociated hippocampal cultures. Individual neurons and their spines were visualized due to transfection with a green fluorescent protein (GFP). The cultures were pre-treated with either DP-b99 (0.12 µM or 20 µM) or GM6001 (25 µM) and then incubated with auto-activating, recombinant MMP-9 for 1 h (see Michaluk et al. [20]). We observed a marked elongation of dendritic spines in MMP-9-treated cultures, whereas DP-b99 significantly prevented the formation of these filopodia-like structures (Fig. 4 A, B). Since the actin cytoskeleton plays a fundamental role in spine morphogenesis and dynamics, we examined F-actin organization within dendrites using Alexa Fluor 568-coupled phalloidin. We found that treatment of the cultures with recombinant MMP-9 induced redistribution of F-actin from spine heads into the dendritic shaft and this effect was abolished by DP-b99 treatment at both applied doses (Fig. 4 A, C).

## Discussion

The major results of this study can be summarized as follows. DP-b99, when administered to animals, suppresses development of chemically kindled seizures and mossy fiber sprouting in the brain. Furthermore, DP-b99 inhibits MMP-9 activity in a biochemical assay and affects such MMP-9-dependent neuronal functions, as kainate excitotoxicity, β-dystroglycan cleavage and morphological modulation of dendritic spines, possibly involved in the synaptic plasticity. Thus, this compound displays a potent role in preventing aberrant plasticity driven byMMP-9.

DP-b99 is a lipophilic, cell permeable derivative of BAPTA, which selectively modulates the distribution of metal ions in a hydrophobic milieu. DP-b99 was demonstrated to attenuate zinc-induced neuronal cell death [5] and showed promise in the treatment of acute stroke patients in a Phase II clinical study [2]. However, recent data has revealed that DP-b99 did not help patients with ischemic stroke in the early-terminated MACSI trial [4]. Lack of evidence that DP-b99 offers any clinical benefit in this trial may suggest that dose and/or application time window was not suitable for protection cells from zinc neurotoxicity. In the current studies we evaluate whether this membrane-activated chelator affects seizure development in PTZ-treated mice. Since pharmacokinetic data is not available for DP-b99 in mice therefore, here, we have used the accessible information in rats [1] to guide the experiments in mice. As for the selection of DP-b99 dosage, we were guided by earlier studies describing the safety and tolerability of DP-b99 in healthy humans [31].

We found a significant delay in the development of PTZ-induced kindled seizures in mice treated with DP-b99, as compared to the vehicle-treated animals. It is known that epileptic seizures and the associated changes in neuronal plasticity are MMP-9-dependent [18] and require augmentation of expression as well as activity of the enzyme [19]. Although it was demonstrated that β-DG can be cleaved extracellularly by MMP-2 and MMP-9 [32], it was reported that enhanced degradation of β-DG produced within minutes after seizures is abolished in MMP-9 KO mice and multiple lines of evidence point to MMP-9 as responsible for β-DG cleavage in activated neurons [24].

In the current study we show that MMP-9 activity is inhibited by DP-b99 and the treatment with this lipophilic metal ion chelator reduces proteolysis of β-DG both *in vitro* and *in vivo*. Wang and Tsirka [33] showed that systemically intraperitoneal administration of broad-spectrum MMPs’ inhibitor (GM6001) reduces the intracerebral hemorrhage-induced gelatinolytic activity by reduction of pro- and active MMP-9 (and pro-MMP-2) proteins levels. Since MMP inhibitors should have neither direct effects on MMP expression nor its activation, the authors suggested that MMP-9 expression and activation might be mediated by other MMPs which might be the direct target of MMP inhibition [33]. Similarly, DP-b99 may act specifically by blocking MMP-9 activity but not influencing other processes in the MMP-9 life cycle.

We found that treatment with DP-b99 had a neuroprotective effect against kainate induced neuronal loss *in vitro*. Kainic acid, an excitotoxic analogue of glutamate, evokes seizure activity followed by histopathological changes including cell death in CA1 and CA3 hippocampal regions [34]. As a result, the granule neurons of the dentate gyrus lose their CA3 targets as well as their synaptic input from the entorhinal cortex and undergo aberrant neuronal plasticity to establish new synaptic connections. This form of plasticity involves elimination of dendritic spines and sprouting of granule cell axons [35], [36], [37]. The essential role of MMP-9 in these phenomena has previously been established [18], [27].

We showed that DP-b99 abolished MMP-9 induced elongation of dendritic spine in cultured hippocampal neurons. However, other findings indicate divergent effects of MMP-9 on spine morphology and raise the question regarding the factors playing the role in apparently opposing effects. In contrast to our observations, studies by Wang et al. [38] provided evidence that MMP-9 was necessary for the enlargement of spines associated with LTP induction in acute hippocampal slices. Furthermore, the MMP-9-driven cleavage of intracellular adhesion molecule-5 (ICAM-5), mainly located on immature spines, was observed following neuronal stimulation and led to spine maturation [39]. On the other hand, Tian et al. [40] have found that ICAM-5 promoted elongation of dendritic spines. This discrepancy presumably results from differences in MMP-9 dose (local vs. bath application), duration of experiment as well as maturity of studied neurons (for review see: [41], [42]).

One should notice that the blocking of MMP-9 activity may exert potential adverse effects, however, not in the case of PTZ treatment. The effect of inhibitors is closely associated with the function performed by protease (detrimental or beneficial). For instance, locally secreted MMP-9 is involved in the structural and functional plasticity of activated synapses [38], [43]. In this case using a molecule that targets MMP-9 may be harmful and may disturb functioning of the neuronal network. Slight increase in MMP-9 activity on synapses can lead to epilepsy and applying the inhibitor has potential anti-epileptic effect. On the other hand, in pathological conditions when secretion of MMP-9 is enhanced after injury, blocking of its activity may be neuroprotective.

Our study provides evidence for DP-b99 exerting protection against kainate-induced neuronal loss *in vitro.* Previously reported data shows DP-b99 at 20 µM to be effective in blocking Zn^2+^ neurotoxicity [5]. Here we find that a lower concentration, 0.12 µM, possibly more physiologically relevant, is more potent. Conditions when a higher concentration of inhibitor causes less inhibitory effect are biologically plausible. Indeed there are many examples of inverted U-shaped dose response curves in the literature. In the case of DP-b99, this phenomenon may be a reflection of its multi-target nature.

We also observed that DP-b99 at 0.12 µM was more effective in the blocking of gelatinolytic activity of MMP-9, while both applied doses of DP-b99, 0.12 µM and 20 µM, exerted similar effect on the dendritic spine morphology. This divergence can result from the different interaction between DP-b99 and MMP-9 in the enzymatic assay with DQ-gelatin compared with such interaction in living neuronal cells. Moreover, our unpublished data showed that in hippocampal culture treated with glutamate the concentration of MMP-9 is about 40 ng/ml, what corresponds to 0.001 µM. So, both DP-b99 doses, 20 µM and 0.12 µM, are beyond the threshold dose and might have equal efficacy on MMP-9 inhibition. Saturation of the drug effect beyond that threshold dose and no dose-effect has been observed for many drugs.

Animal studies showed that DP-b99 might prevent the development of PTZ-kindled seizures. Although MMP-9 can be the immediate target for DP-b99 accounting for the observed changes in PTZ-kindled animals, there are still remaining questions about the specificity and mechanisms of this interaction during PTZ-induced seizures. Since DP-b99 has been developed as a multi-target agent to selectively chelate different divalent metals, such as calcium, zinc and copper; it is difficult to define unequivocally the target of the chelator *in vivo*. For instance, experimentally-induced status epilepticus results in damage to the hippocampus and other temporal lobe structures. As has been shown previously, proinflammatory cytokines such as interleukin-1β, TNF-α are up-regulated in both neurons and glia in the brain structures generating seizures [44], [45]. Moreover, increased susceptibility to seizures was observed in rats with TNF-α injection, and to the contrary, a blocker of TNF receptor-1 had potent antiepileptic activity [46]. It is known that MMPs might be regulated by cytokines, particularly TNF-α [47]. It has been reported that DP-b99 treatment blocked basal activation of MMP-9 and reduced TNF-α-induced expression in primary cultured glial cells [1]. Although we did not check the level of TNF-α, we can speculate that DP-b99 may not only affect MMP-9, but also may have a synergistic effect by lowering TNF-α action on MMP inhibition.

In conclusion, our data show that DP-b99 affects important hallmarks of epileptogenesis, such as neuronal cell loss and aberrant synaptic plasticity by the remaining cells. Therefore, one may consider that pharmacological inhibitors of MMPs represent possible candidates for anti-epileptogenic therapies. However, more studies should be directed towards the specific role of DP-b99 in preventing epilepsy.
